# Balance factors affecting the quality of life in patients with knee osteoarthritis

**DOI:** 10.4102/sajp.v78i1.1628

**Published:** 2022-03-30

**Authors:** Tian-Shyug Lee, Hsiang-Chuan Liu, Shih-Pin Lee, Yi-Wei Kao

**Affiliations:** 1 Graduate Institute, Faculty of Business Administration, Fu Jen Catholic University, New Taipei City, Taiwan

**Keywords:** osteoarthritis, balance control, steadiness, limit of stability, quality of life, sway velocity, physical health component, mental health component

## Abstract

**Background:**

Knee osteoarthritis (OA) affects the quality of life (QOL) and balance control of elderly people; our study explored the balance factors that affected the QOL in patients with knee OA.

**Objectives:**

To determine the balance factors that affected the QOL of patients with knee OA who attended general clinics.

**Method:**

A total of 30 healthy controls and 60 patients with mild-to-moderate bilateral knee OA, all aged 55–75 years, were enrolled in our cross-sectional study. All participants were interviewed; the Medical Outcomes Study 36-Item Short-Form Health Survey was used to assess their QOL in eight dimensions, and the Balance Master System was used to evaluate their balance control according to six parameters. Descriptive statistics were used to reduce the data; an independent *t*-test determined differences between the two groups, and a multiple regression analysis was undertaken to establish associations between variables from the balance control test and SH36 physical and mental health components. The level of statistical significance was set at 5%.

**Results:**

In the OA group, significant negative correlations were observed between sway velocity and the physical health component (*p* = 0.003) and between sway velocity and the mental health component (*p* = 0.006). Thus, sway velocity had a major impact on the QOL of patients with knee OA.

**Conclusions:**

The sway velocity at the centre of gravity in balance control was a crucial factor for determining the QOL of patients with bilateral knee OA.

**Clinical implications:**

Sway velocity is a key factor affecting the QOL and may provide a basis to formulate preventive actions and design treatment goals for patients with knee OA.

## Introduction

With the rapid increase in the ageing population, the risk of chronic diseases in older adults has also increased. Osteoarthritis (OA), dementia, stroke and coronary heart disease are the major diseases that cause disability in older adults (Dina et al. 2020; Wanless 2006). Osteoarthritis, a common and chronic progressive disease, not only causes pain in the lower limb joints but also worsens quality of life (QOL), particularly in those aged > 50 years.

The prevalence of knee OA is estimated to increase by 40% in 2025 because of the ageing of the world population (Farr, Miller & Block 2013). According to USA statistics, 70% – 90% of older adults with knee OA experience pain and discomfort (Oliveria et al. 1995). The pain and restricted range of motion of the leg caused by knee OA adversely affect the balance and QOL of older adults.

Many aspects of the human body, such as the vestibular system, vision, muscular strength and cognition, are related to balance control, which is an important ability in daily life. Maintaining balance is crucial in daily activities. Balance is defined as ‘the ability of a person to maintain the centre of gravity within a certain range or support area (Nashner 1993)’. This ability is crucial for maintaining maximal stability in the standing position with a support base that lies within the range of the contact area of the bilateral feet (Karen et al. 2005; Nashner 1993). There are many clinical balance tests, such as the timed up and go test, 10-m walking test and the Berg balance scale, which are considered to have high intraclass correlation and reliability between test–retests.

However, these tests may have subjective factors depending on the examiner and subject, and they cannot assess all aspects of balance control (Kim et al. 2011). Murray, Seireg and Sepic (1975) used force plates to assess balance ability and they indicated the two major characteristics of balance as being steadiness and limit of stability. Steadiness refers to the slight swaying of the body during the maintenance of a static standing posture. A smaller degree of sway reflects greater steadiness and vice versa. The limit of stability or maximum voluntary excursions refers to the maximum inclination angle at which the human body actively tilts forward, backward, left and right to maintain balance and avoid falling. A greater angle of inclination corresponds to a higher limit of stability (Lim & Lee 2012; Murray et al. 1975).

Disease progression in patients with knee OA results in restricted physiological functions because of limited recovery from pain and a lack of normal knee function. The daily activities of these patients are consequently increasingly affected, thus reducing their capacities for labour management, leisure and social activities and their sleep quality and in turn affecting their QOL (Sutbeyaz et al. 2007). Therefore, QOL is crucial for evaluating patients with knee OA. In general, QOL is assessed to determine the effect that a disease has on patients.

The World Health Organization defines QOL as individuals’ perception of their position in life in the context of the culture and value systems in which they live and in relation to their goals, expectations, standards and concerns (Ackerman et al. 2014). The Medical Outcomes Study 36-Item Short-Form Health Survey (SF-36) is short, easy to complete and comprehensible. In addition, it is a universal assessment tool that can be used to determine the QOL of patients with knee OA in relation to other health conditions and psychological and social factors (Kawano et al. 2015).

Many studies have been conducted on balance control (Hyoungjin & Taewoon 2018; Lim & Lee 2012; Nafiseh et al. 2014; Pua et al. 2011) and QOL (Ackerman et al. 2014; Kawano et al. 2015; Sutbeyaz et al. 2007) of patients with knee OA, but few have investigated the relationship between balance control and QOL in patients with knee OA. The aim of our study was therefore to determine the balance factors affecting the QOL in patients with knee OA and to find the balance factors that affected their QOL by evaluating the subjects’ objective balance control.

## Method

**FIGURE 1:**
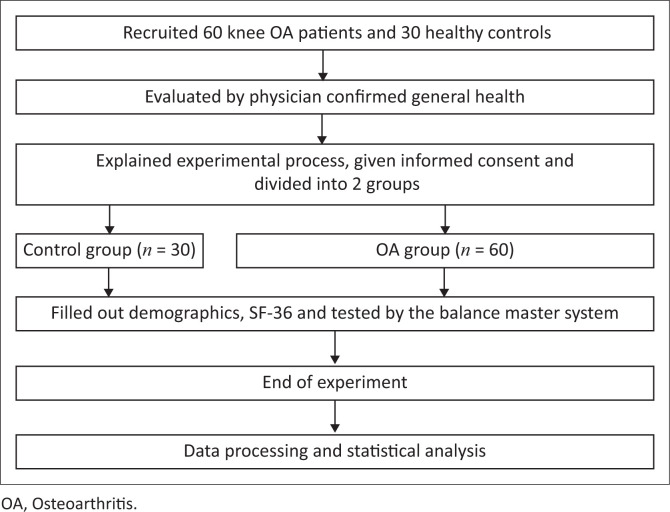
Research design.

This cross-sectional study (Figure 1 shows the flow diagram) included a sample of 60 elderly patients aged 55–75 years with mild-to-moderate bilateral knee OA (grade 2 or 3 on the Kellgren–Lawrence scale [K-L scale]) treated in a general rehabilitation clinic. Patients who met our study’s inclusion criteria were recruited through simple random sampling, and 30 age-matched healthy controls were also recruited. Some controls were selected after responding to the advertisement for our study. Additional controls were recruited from the families of patients who visited the clinic during our study period.

The same physician confirmed knee OA diagnosis through radiography (weight-bearing anteroposterior, lateral and skyline views) by using the K-L scale and assisted in confirming the general health of the enrolled participants. On the K-L scale: (1) grade 0: no characteristics of OA, (2) grade 1: suspected knee OA, characterised by narrowing joint space and possible osteophytes, (3) grade 2: mild knee OA, with small osteophytes and possible narrowing knee joint, (4) grade 3: moderate knee OA, characterised by multiple moderate-sized osteophytes, definite joint space narrowing and possible deformation of the bone ends, (5) grade 4: severe knee OA, characterised by multiple large osteophytes, severe joint space narrowing, marked sclerosis and definite bony end deformity (Nafiseh et al. 2014).

Patients were excluded if they had lower limb nerve pain, muscular or skeletal injuries or lesions, vision or inner ear vestibule disorders or proprioceptive nerve damage or lesions that affected their balance in the past 6 months. Moreover, patients were excluded if they had severe knee OA that caused them difficulty in standing, ongoing participation in other studies or other major injuries or illnesses that would affect our study, such as grade 2 or higher cardiopulmonary function diseases, neurological abnormalities, cardiopulmonary failure or a history of stroke. Controls reported no current or past lower limb pain, the physical examination of both knees were normal and the self-reported history of vertigo, stroke or other conditions that might impair balance were excluded.

## Procedure

Basic demographic and QOL information were obtained from all participants through the SF-36. Demographic characteristics included sex, age, height and weight. The SF-36 is valid and reliable (the overall Cronbach’s alpha coefficient is 0.883; Berliana et al. 2021) and consists of two major components, each of which has four dimensions, namely the physical health component (four dimensions: physical function, body role limitations because of physical health problems, body pain and general health problems) and the mental health component (four dimensions: vitality, social function, emotional status and/or role limitations because of emotional health problems and general mental health problems). Eight dimensions were evaluated in total, and for each of the dimensions, we obtained a score after applying a measurement scale with values from 0 (worst health status) to 100 (best health status) (Kawano et al. 2015).

**FIGURE 2:**
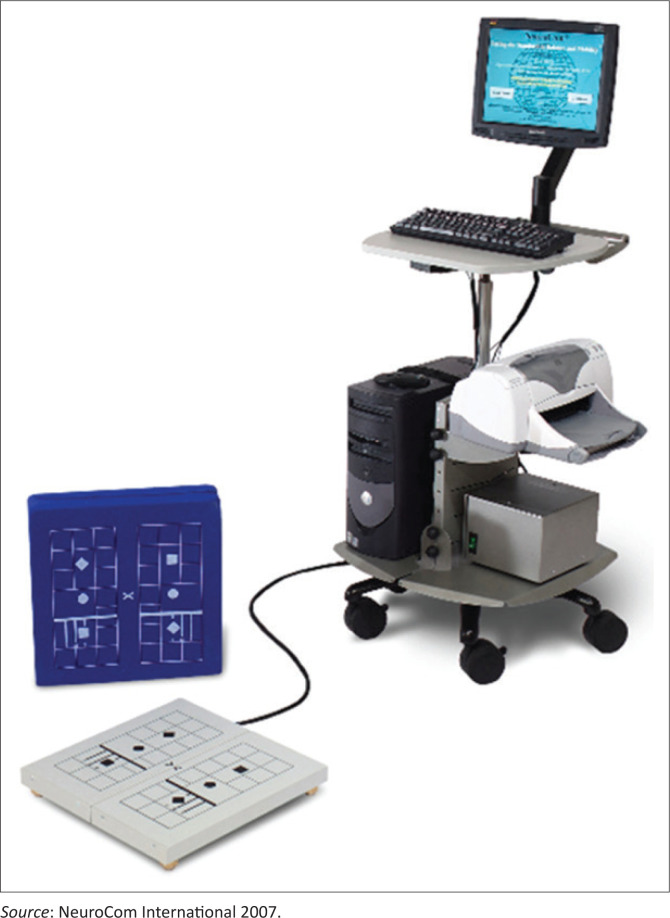
NeuroCom’s® Balance Master®.

The Balance Master System (Neurocom Inc., OR, USA) has moderate to high reliability (intraclass correlation coefficient range: 0.78−0.91) and has acceptable predictive validity (*r*^2^ range: 0.15−0.17) (Chi-Wen et al. 2007) and was used to assess the balance control of the participants (NeuroCom International 2007). The system comprises a force measuring platform and a computer to quantify body swing and measure the position of the centre of gravity related to the supporting foundation. The platform consists of two 9 × 60 in.^2^ force plates. The computer (version 7.0 software) was placed in front of the platform, and the height of the screen was aligned to the eye level of the participants (Figure 2). This system was used to measure steadiness with the Modified Clinical Test of Sensory Interaction on Balance and to measure the limit of stability with the Limits of Stability Test.

The Modified Clinical Test of Sensory Interaction on Balance measures the sway velocity (SV) of the centre of gravity under four conditions: (1) standing on a stable base with eyes open, (2) standing on a stable base with eyes closed, (3) standing on an unstable (foam) base with eyes open and (4) standing on an unstable (foam) base with eyes closed (Sghaier et al. 2020). Each condition has three trials lasting 10 s each. The SV of the centre of gravity was the ratio of the degrees travelled by the centre of gravity to the duration (seconds) of the test.

In the limits of stability test, the participants shift their weight to move a cursor projected on the computer screen to one of eight targets surrounding a centre starting position: (1) forward, (2) forward-right, (3) forward-left, (4) right, (5) right-backward, (6) backward, (7) left and (8) left-backward. Furthermore, the participant is instructed to move the cursor to each appropriate target as directly, quickly and accurately as possible within 8 s. On the basis of eight trials of the limits of stability test, five parameters were calculated according to the reaction time (RT), which was the time from the signal moving to the participant’s first moving; the centre of gravity movement velocity (MV), which was the average speed of the centre of gravity movement; endpoint excursion (EPE), which was the farthest on-axis distance the centre of gravity reached by the end of the first sustained centre of gravity excursion towards the test target, expressed as a percentage; maximum excursion (MXE), which was the farthest displacement towards the designed target reached by the centre of gravity during the test, expressed as a displacement percentage and direction control (DC), which was a comparison between the amount of movement in the intended direction and the amount of extraneous movement, expressed as a percentage (Hyoungjin & Taewoon 2018). To eliminate the influence of shoes, the participants were asked to perform the balance test whilst barefoot.

We used the mean values of six parameters obtained from the Balance Master System separately to compare with the SF-36 results to determine the relationship between balance control and QOL in the control and OA groups. We then compared balance control with the physical and mental health components of the SF-36 to further determine the balance factor affecting the QOL in patients with knee OA.

## Statistical analysis

All statistical analyses were performed in R software (version 3.6.1; R Foundation for Statistical Computing, Vienna, Austria). Descriptive statistics were generated for the demographic characteristics in the OA and control groups. Furthermore, the mean and standard deviation of the age, height and weight were assessed and presented as medians and then frequencies and percentages for the sex of the participants. We used an independent *t*-test to determine the differences between the two groups in the six parameters obtained from the balance control test and eight dimension scores of SF-36. A multiple regression analysis was performed to evaluate the association of the six parameters from the balance control test with the SF-36, physical health component and mental health component in the two groups. For all analyses, the level of statistical significance was set at 5%.

### Ethical considerations

Informed consent was obtained from the participants before the study was conducted. This study received approval from the Ethics Committee of Fu Jen Catholic University (FJU-IRB NO: C107179).

## Results

In total, 90 individuals participated in the study (control group [30], OA group [60]). The mean age of the control group and OA group was 66.40 and 66.28 years old; the mean height of the control group and OA group was 162.030 and 160.400 cm, and the mean weight of the control group and OA group was 70.03 cm and 69.330 cm, respectively. A comparison of demographic characteristics between the control and OA groups is presented in Table 1. No significant difference was observed between the two groups in terms of the sex (*p* = 0.940), age (*p* = 0.920), height (*p* = 0.249) or weight (*p* = 0.713).

**TABLE 1:** Demographic characteristics of patients in the control and osteoarthritis groups.

Demographic characteristics	Control group (*n* = 30)		OA group (*n* = 60)		*p*
	*N*	%	*n*	%	
**Sex**	-	-	-	-	0.940
Male	17	56.7	32	53.3	-
Female	13	43.3	28	46.7	-
Age (years)	66.40	5.48	66.28	4.98	0.920
Height (cm)	162.03	6.11	160.40	6.38	0.249
Weight (kg)	70.03	8.62	69.33	8.42	0.713

Note: Values for age, height and weight are expressed as the mean (standard deviation).

OA, osteoarthritis.

Statistically significant mean difference (*p* < 0.05).

Table 2 presents the results of the QOL assessment between the two groups based on the SF-36. Amongst the eight dimensions (PF, BR, BP, GH, VT, SF, ES, MH), the scores of control group were better than that of the OA group. For all variables, there were statistically significant differences observed between the two groups (*p* < 0.001), indicating superior physical and mental health of the control group participants.

**TABLE 2:** Mean and standard deviation of the eight dimensions of the SF-36 in the control and osteoarthritis groups.

Components	Dimensions	Control group (*n* = 30)		OA group (*n* = 60)		*p*
		Mean	Standard deviation	Mean	Standard deviation	
Physical health component	PF	91.250	11.54	36.50	24.28	< 0.001
	BR	84.58	23.31	28.33	24.99	< 0.001
	BP	82.08	26.40	17.29	19.81	< 0.001
	GH	75.50	15.50	36.83	21.35	< 0.001
Mental health component	VT	68.54	17.17	41.25	19.23	< 0.001
	SF	87.92	15.22	36.46	32.81	< 0.001
	ES	72.22	27.88	28.75	26.81	< 0.001
	MH	69.26	14.35	44.44	24.33	< 0.001

PF, physical function; BR, role limitations due to physical health problems; BP, body pain; GH, general health problems; VT, vitality; SF, social function; ES, role limitations due to emotional health problems; MH, general mental health problems.

Statistically significant mean difference (*p* < 0.05).

Considering steadiness during the balance control assessment (Table 3), based on the Modified Clinical Test of Sensory Interaction on Balance, the values of SV of the centre of gravity of the OA and control groups were 0.870 ± 0.24° per second and 0.730 ± 0.22° per second, respectively. The SV of the centre of gravity was significantly different between the groups (*p* = 0.011). The results implied that the OA group had more sway and less stability in a standing position.

**TABLE 3:** Mean and standard deviation of the six parameters of balance control in the control and osteoarthritis groups.

Balance control test	Balance parameters	Control group (*n* = 30)		OA group (*n* = 60)		*p*
		Mean	Standard deviation	Mean	Standard deviation	
MCTSIB	SV (degrees per second)	0.730	0.22	0.870	0.24	0.011
Limits of stability test	RT (seconds)	0.970	0.20	1.080	0.19	0.016
	MV (degrees per second)	3.240	1.49	3.730	0.98	0.066
	EPE (%)	65.700	9.88	70.600	11.16	0.045
	MXE (%)	85.170	7.86	78.120	12.24	0.005
	DC (%)	74.930	7.21	66.870	8.80	< 0.001

OA, osteoarthritis; SV, sway velocity; RT, reaction time; MV, movement velocity; EPE, endpoint excursion; MXE, maximum excursion; DC, directional control; MCTSIB, the modified clinical test of sensory interaction on balance.

Statistically significant mean difference (*p* < 0.05).

Regarding the limit of stability with the Limits of Stability Test (Table 3), the RT, MV, EPE, MXE and DC values of the OA and control groups were 1.08 ± 0.19 seconds and 0.97 ± 0.2 seconds, 3.73 ± 0.98° per second and 3.24 ± 1.49° per second, 70.60% ± 11.16% and 65.70% ± 9.88%, 78.12% ± 12.24% and 85.17% ± 7.86% and 66.87% ± 8.8% and 74.93% ± 7.21%, respectively. The groups differed significantly in terms of the RT (*p* = 0.016), EPE (*p* = 0.045), MXE (*p* = 0.005) and DC (*p* < 0.001). The results indicate that the OA group had a longer RT, higher EPE and lower MXE and DC than did the control group. Regarding MV (*p* = 0.066), no significant difference was observed between the two groups.

Table 4 demonstrates the results of the multiple regression analysis regarding the associations of the six parameters of balance control with the average score of SF-36 in eight dimensions in the control and OA groups. In the control group, no statistically significant difference was observed between the balance control and the SF-36 (SV, *p* = 0.776; RT, *p* = 0.403; MV, *p* = 0.633; EPE, *p* = 0.318; MXE, *p* = 0.771 and DC, *p* = 0.898). In the OA group, only SV (*β* = −0.39, *p* = 0.003) was significantly associated with the SF-36, exhibiting a negative correlation, whereas the RT (*p* = 0.177), MV (*p* = 0.129), EPE (*p* = 0.564), MXE (*p* = 0.052) and DC (*p* = 0.057) were not associated with the SF-36.

**TABLE 4:** Multiple regression analysis of the associations of the six parameters of balance control with the SF-36 in the control and osteoarthritis groups.

Balance control Test	Balance parameters	Control group (*n* = 30)					OA group (*n* = 60)				
		Unstandardised coefficients			*β*	*p*	Unstandardised coefficients			*β*	*p*
		B	95%	CI			B	95%	CI		
MCTSIB	SV	−4.026	−32.888	24.837	−0.080	0.776	−31.243	−51.559	−10.927	−0.390	0.003
Limits of Stability Test	RT	16.136	−23.018	55.290	0.292	0.403	17.105	−7.986	42.197	0.173	0.177
	MV	−1.062	−5.607	3.482	−0.146	0.633	4.006	−1.209	9.221	0.208	0.129
	EPE	−0.254	−0.769	0.261	−0.231	0.318	0.138	−0.339	0.616	0.082	0.564
	MXE	0.121	−0.730	0.972	0.088	0.771	−0.432	−0.869	0.004	−0.280	0.052
	DC	0.048	−0.727	0.824	0.032	0.898	0.562	−0.018	1.142	0.262	0.057

OA, osteoarthritis; B, regression coefficient; CI, confidence interval; *β*, standardised coefficient; SV, sway velocity; RT, reaction time; MV, movement velocity; EPE, endpoint excursion; MXE, maximum excursion; DC, directional control; MCTSIB, the modified clinical test of sensory interaction on balance.

Statistically significant mean difference (*p* < 0.05).

We further separately compared the balance control with the average score of physical and mental health components. The association between the balance control and physical health components is presented in Table 5. In the control group, no statistically significant difference was observed between the balance control and the physical health component (SV, *p* = 0.653; RT, *p* = 0.357; MV, *p* = 0.321; EPE, *p* = 0.112; MXE, *p* = 0.361 and DC, *p* = 0.856). In the OA group, only SV (*β* = −0.401, *p* = 0.003) was significantly associated with the physical health component, exhibiting a negative correlation, whereas RT (*p* = 0.416), MV (*p* = 0.273), EPE (*p* = 0.602), MXE (*p* = 0.068) and DC (*p* = 0.131) were not associated with the physical health component.

**TABLE 5:** Multiple regression analysis of the associations of the six parameters of balance control with physical health component of the SF-36 in the control and osteoarthritis groups.

Balance control test	Balance parameters	Control group (*n* = 30)					OA group (*n* = 60)				
		Unstandardised coefficients			*β*	*p*	Unstandardised coefficients			*β*	*p*
		B	95%	CI			B	95%	CI		
MCTSIB	SV	−7.658	−42.446	27.130	−0.116	0.653	−29.674	−48.847	−10.501	−0.401	0.003
Limits of Stability Test	RT	21.445	−25.747	68.638	0.297	0.357	9.678	−14.001	33.357	0.106	0.416
	MV	−2.684	−8.162	2.794	−0.282	0.321	2.717	−2.205	7.638	0.153	0.273
	EPE	−0.496	−1.117	0.124	−0.345	0.112	0.118	−0.333	0.569	0.075	0.602
	MXE	0.462	−0.563	1.488	0.256	0.361	−0.382	−0.794	0.030	−0.268	0.068
	DC	−0.083	−1.018	0.852	−0.042	0.856	0.419	−0.129	0.966	0.211	0.131

B, regression coefficient; CI, confidence interval; *β*, standardised coefficient; SV, sway velocity; RT, reaction time; MV, movement velocity; EPE, endpoint excursion; MXE, maximum excursion; DC, directional control; MCTSIB, the modified clinical test of sensory interaction on balance.

Statistically significant mean difference (*p* < 0.05).

The associations between the balance control and the mental health component are presented in Table 6. In the control group, no statistically significant difference was observed between the balance control and the mental health component (SV, *p* = 0.980; RT, *p* = 0.615; MV, *p* = 0.823; EPE, *p* = 0.966; MXE, *p* = 0.638; DC, *p* = 0.673). In the OA group, SV (*p* = 0.006) and DC (*p* = 0.036) were significantly associated with the mental health component, with SV (*β* = −0.359) exhibiting a negative correlation and DC (*β* = 0.288) exhibiting a positive correlation; however, RT (*p* = 0.090), MV (*p* = 0.079), EPE (*p* = 0.560) and MXE (*p* = 0.056) were not associated with the mental health component.

**TABLE 6:** Multiple regression analysis of the associations of the six parameters of balance control with the mental health component of the SF-36 in the control and osteoarthritis groups.

Balance control test	Balance parameters	Control group (*n* = 30)					OA group (*n* = 60)				
		Unstandardised coefficients			*β*	*p*	Unstandardised coefficients			*β*	*p*
		B	95%	CI			B	95%	CI		
MCTSIB	SV	−0.393	−32.763	31.977	−0.008	0.980	−32.812	−55.882	−9.742	−0.359	0.006
Limits of Stability Test	RT	10.827	−33.086	54.739	0.189	0.615	24.532	−3.960	53.025	0.218	0.090
	MV	0.559	−4.538	5.656	0.074	0.823	5.296	−0.626	11.218	0.241	0.079
	EPE	−0.012	−0.590	0.566	−0.011	0.966	0.159	−0.384	0.701	0.082	0.560
	MXE	−0.220	−1.174	0.735	−0.153	0.638	−0.482	−0.978	0.013	−0.273	0.056
	DC	0.180	−0.690	1.050	0.115	0.673	0.706	0.047	1.364	0.288	0.036

B, regression coefficient; CI, confidence interval; *β*, standardised coefficient; SV, sway velocity; RT, reaction time; MV, movement velocity; EPE, endpoint excursion; MXE, maximum excursion; DC, directional control; MCTSIB, the modified clinical test of sensory interaction on balance.

Statistically significant mean difference (*p* < 0.05).

## Discussion

People must constantly change their posture to perform daily activities; therefore, they need to control their centre of gravity on the supporting base to maintain balance. Balance control includes maintaining static postures (steadiness) and complex dynamic movements (limit of stability), which are particularly essential in daily life activities. Knee OA has major effects on the daily life and mobility of older adults, and it is related to their independence and health-related QOL. Knee OA is a common degenerative disease in older adults that causes pain, stiffness and dysfunction. Many studies have shown that knee OA causes a decrease in QOL (Ackerman et al. 2014; Kawano et al. 2015; Maiara et al. 2020; Sutbeyaz et al. 2007). Furthermore, it reduces the patient’s balance control (Hyoungjin & Taewoon 2018; Lim & Lee 2012; Nafiseh et al. 2014; Pua et al. 2011). A study on knee OA patients and controls without knee OA in Taiwan, showed that postural stability is related to QOL (Hsieh et al. 2013).

We compared the two groups’ balance control test and the QOL assessment. The results revealed that only SV was significantly associated with QOL in the OA group, exhibiting a negative correlation. To further explore the relationship of balance control with physical and mental health components, we analysed the associations of the balance control test with the physical and mental health components in the OA group and compared them with the control group. The results revealed a significant negative association of SV with the physical and mental health components. A comparison of balance control and the mental health component in the OA group revealed that SV is negatively and DC is positively correlated with the mental health component. Statistical analysis indicated that SV has a greater impact than DC on the mental health component (Table 6, *β*: 0.359 > 0.288).

In the OA group, SV was significantly negatively correlated with QOL, meaning that a lower QOL score corresponded to more swaying and less stability in a standing position. Overall, the SV of the centre of gravity was a crucial factor influencing QOL.The SV of a painful leg when standing was greater than that of a healthy leg, which meant that the knee OA group had more sway and was less stable when standing (Lim & Lee 2012). A recent study of patients with Parkinson disease used the force measuring platform to measure SV and the relationship between QOL. The results also showed a significant negative correlation between the measures of SV and QOL (Deborah et al. 2020). Thus, increased SV is associated with reduced QOL.

A study on the balance of Tai Chi exercise on chronic stroke patients (Kim, Kim & Lee 2015) and another study on the effect of karate training on the QOL and balance control of the elderly (Chateau-D et al. 2010) have shown that the SV was reduced, and physical and mental health were improved after training. Furthermore, studies have explored the possibility of improving balance parameters (e.g. SV) through exercise training; both physical and mental health components showed improvements in SF-36 scores after exercise training (Chateau-D et al. 2010; Kim et al. 2015). Therefore, future research should analyse the relationship between balance control and QOL of patients with knee OA before and after exercise or treatment.

However, mental health is often overlooked as a crucial factor for dysfunction in people with knee OA. One study revealed an association between the deterioration of mental health and OA risk (Wise et al. 2010). Furthermore, Gonzalo et al. stated that SV is related to mental health in patients with stroke (Gonzalo, Lakshmi & Tanvi 2020). In our study, SV was not only negatively correlated with the physical health component of patients with knee OA but also had a significant negative correlation with the mental health component.

Maintaining balance control requires a certain amount of attention and an individual’s performance in this respect depends on the complexity of the posture task: the more challenging the posture task, the greater the amount of attention required. Furthermore, studies have shown that declines in mental functioning can reduce the activity of the prefrontal cortex and anterior cingulate cortex (Gonzalo et al. 2020; Holtzer et al. 2011; Lajoie et al. 1993), which are the two crucial brain regions involved in executive functions. They are important for gait pattern and balance control.

Relevant studies have suggested that because balance is necessary for performing daily activities, understanding the impact of knee OA on the postural swing (SV) can not only facilitate the development of more effective treatments but also help clarify the adverse mechanisms of knee OA (Hinman et al. 2002). Balance training can improve stability and self-confidence and safely increase physical activity (Heon-Gyu, Jungae & Byoung-Hee 2021). Studies show that some balance training, for example, to stand up from a chair and raise heels when standing, use a step box to go up and down steps, walk around a cone, practice tiptoe gait and change direction whilst walking are effective in improving body function, balance and QOL (Ayelet Dunsky 2019; Heon-Gyu et al. 2021; Madureira et al. 2007). Therefore, we suggest that balance training has a positive effect in promoting body function, balance and QOL of patients with knee OA.

Our study demonstrated several methodologic strengths. We combined objective functional performance (balance control assessment) and subjective assessment (SF-36) to determine the balance factors that affected the QOL in patients with knee OA. These analyses included knee OA patients and healthy control participants. Participants were sampled from general clinics in the community and evaluated and measured.

## Study limitations

Our study only included patients from general clinics; therefore, the results may not be generalisable. Moreover, our study is limited by its small sample size. However, our goal was to determine the balance factors affecting QOL in patients with knee OA. It may provide a basis to formulate preventive measures for healthy individuals and design treatment goals for patients with knee OA. We used the Chinese versions of SF-36 for QOL assessment. Although translated instruments provide a reliable method of assessing QOL in cross-cultural contexts, the influence of cultural differences and their possible confounding effects must be acknowledged.

## Conclusion

Our study sufficiently demonstrated that SV is a key factor affecting QOL and may provide a basis for healthy people to formulate preventive actions and design treatment goals for patients with knee OA. For earlier interventions of patients with knee OA and future related research, our study provides the following recommendations:

- The primary goal of therapy for knee OA is to improve patients’ QOL, such as their vitality, positive emotions and social function, in addition to mitigating their symptoms and improving their physical function.
- The evaluation of treatment effectiveness for patients with knee OA should include both balance control evaluation and objective QOL measures, such as the SF-36.
- To extend the findings of our study, future research should analyse the balance control and QOL of patients with knee OA before and after treatment and evaluate the effectiveness of treatment prescriptions in improving balance control.
